# Practice Areas, Skills, and Core Competencies of Advanced Practice Physiotherapists Working in Tertiary Care in Germany: Protocol for a 3-Round Delphi Study to Establish Expert Consensus

**DOI:** 10.2196/90704

**Published:** 2026-06-11

**Authors:** Lukas Kühn, Leonie Heimken, Lena Frenz, Annalena Paus

**Affiliations:** 1 Department of Therapeutic Health Professions, University Hospital Münster, Albert-Schweitzer-Campus 1, Münster, North Rhine-Westphalia, 48149, Germany, 49 2518348160

**Keywords:** advanced clinical practice, Delphi technique, inpatient care, acute care, physical therapy

## Abstract

**Background:**

Specifically in primary musculoskeletal care settings, the employment of advanced physiotherapy practice (APP) roles seems to be safe, as well as clinically and financially effective. In tertiary care settings, the implementation of APP roles is still in its infancy. A structured identification of relevant APP roles and a definition of practice areas, needed skills, and core competencies is widely pending.

**Objective:**

This study aims to identify and prioritize the most relevant areas of tertiary care for the implementation of APP roles within the German health care system. Additionally, it is aimed to achieve expert consensus on practice areas, needed skills, and core competencies relevant to execute identified roles.

**Methods:**

A total of 100 national experts will be invited to participate in a 3-round Delphi survey. Participants will purposively be sampled among physiotherapists with extensive clinical and/or academic experiences in German tertiary care. Data will be pseudonymized and collected via online questionnaires. In Delphi round 1, close-ended questions on relevant APP roles and open-ended input on practice areas, skills, and core competencies will be queried. Open-ended questions will be structured according to key domains of the National Health Service multiprofessional framework for advanced clinical practice. In round 2, participants will rate close-ended items on practice areas, skills, and core competencies that emerged from open-ended questions of round 1. Each item will be rated on a 7-point Likert scale ranging from “not relevant at all” to “extremely relevant.” Consensus will be set on ≥70% agreement per item (including “5=fairly relevant,” “6=very relevant,” and “7=extremely relevant”). Items with agreements of ≤30% will be excluded. Items of 31% to 69% agreement will be reevaluated in round 3. Descriptive statistics (median, IQR, and absolute and relative frequencies) will be used to visualize response patterns, and McNemar and Wilcoxon signed-rank tests will be applied to explore changes between rounds.

**Results:**

Data collection took place between January and May 2026. For the first Delphi round, 98 experts were invited, of which 37 fully completed round 1. In round 2, these 37 experts were invited, of which 34 fully completed Delphi round 2. Following these response rates, we were confident to receive the targeted study sample of 20 experts after data collection of Delphi round 3 was completed. Data collection for the third and final round was completed in May 2026 and included 32 final respondents.

**Conclusions:**

Findings will support evidence-based APP role development and contribute to optimizing tertiary care areas relevant to physiotherapy care in Germany.

## Introduction

### Background

Germany’s demographic shift toward an aging population is expected to present significant challenges to its health care system. A rise of chronic diseases, multimorbidity, and the need for long-term nursing care, alongside a simultaneous shortage of professionals in medicine, nursing, and allied health professions, is already forcing relevant stakeholders to discuss new professional roles and care pathways. By 2030, demographic projections indicate that around one-third of Germany’s population will be aged ≥65 years, a development that will significantly increase the prevalence of multimorbidity within the population [1]. At the same time, a shortage of approximately 30,000 to 50,000 physicians [2] and 280,000 to 690,000 nurses [3] is anticipated by 2050. One-third of currently practicing allied health professionals are contemplating a transition to an alternative career path [4]. The main reasons for job dissatisfaction are low salaries, limited career prospects, excessive paperwork, and time constraints [4]. For instance, the German physiotherapy sector is already facing a severe staffing crisis, as merely 8 out of 1000 vacant positions are currently being filled [5].

In physiotherapy-related care settings, the development and implementation of advanced physiotherapy practice (APP) represent a promising strategy to strengthen care pathways in areas at risk of health care undersupply, while simultaneously enhancing the profession’s attractiveness through the creation of new clinical career trajectories. Following the definition of the World Physiotherapy Association, APPs are highly specialized experts who are able to perform complex decision-making in a specific clinical area under risk and unpredictability [6]. In this capacity, they contribute to the integration of evidence-based approaches into clinical practice and take on additional responsibilities in education, research, workforce development, and quality management [6]. This rationale is supported globally; a survey of World Physiotherapy member organizations identified that the primary facilitators for APP development are the need to reduce health care costs, improve efficiency, and respond to the lack of physician capacity [7].

Empirical literature supports the effectiveness of clinically working APPs for patient care. A number of published evidence syntheses highlight that APPs present similar skills as physicians in diagnostic and surgical triage capabilities of musculoskeletal disorders while producing greater pain and disability reductions compared to usual care [8-11]. APP models seem to be more cost-effective compared to usual care in clinical settings of primary, emergency, and specialized secondary care [12]. For instance, mean costs per patient in primary and emergency care are estimated to be around €140 (€1=US $1.64 as of June 2, 2026) lower compared to usual care [12]. Moreover, in orthopedic care, physiotherapist-led triage models for musculoskeletal conditions are associated with reduced waiting times and improvements in patient satisfaction compared to usual care [13].

Outside of orthopedic and emergency settings, both the conceptual definition of APP roles and the systematic empirical assessment of their clinical impact and value to patient care remain insufficiently established. By contrast, the implementation of advanced practice nurses (APNs) has been subject to extensive empirical investigations. A systematic review by Kreeftenberg et al [14] demonstrated that, in intensive care units, APNs achieve comparable quality of care measured by length of hospital stays and mortality. Moreover, care provided by APNs is associated with reduced costs, lower hospital readmission rates, increased patient satisfaction, and other patient-relevant outcome measures [15]. Building on robust evidence supporting the clinical and economic value of APNs in tertiary care, there is a growing rationale for extending advanced practice concepts to other professional groups such as physiotherapy. The development of clearly defined practice areas, tasks, and core competencies represents a critical first step in operationalizing these roles within tertiary care settings and evaluating their context-specific impact.

### Objectives

The primary aim of this Delphi study is to identify and prioritize the most relevant areas of tertiary care for the implementation of APPs within the German health care system. Secondarily, the author group seeks to establish a consensus on needed skills, core competencies, and task delegations required to effectively perform these roles in tertiary care settings. On the basis of these objectives, the research questions we aim to answer are stated as follows: (1) Which practice areas offer the greatest potential for the integration of APP in tertiary care settings in Germany? (2) For each identified practice area, what skills and competencies are critical for effective advanced physiotherapy practice?

## Methods

### Design

This study will follow a 3-round Delphi technique to elicit expert consensus on practice areas, skills, and core competencies of APPs working in tertiary care in Germany. Considering the constructivist nature of this method, we will use rigorous reporting standards aligning with the *Recommendations for the Conducting and Reporting of Delphi Studies* to ensure a high level of replicability [16]. This study has been registered on the open science framework [17].

### Expert Selection and Recruitment

To ensure a high level of expertise and contextual relevance, we will select eligible participants using a purposive sampling approach. Invitations to study participation will be sent via email and/or by telephone. For panel composition, we will recruit experienced physiotherapists who (1) present extensive experience in tertiary care settings, (2) hold a form of leadership position within physiotherapy departments at German tertiary care settings, or (3) are actively engaged in physiotherapy-related research with a clear focus on tertiary care settings. Therefore, selection criteria will include substantial professional experience in tertiary care, advanced academic qualifications, and involvement in clinical, educational, or research activities relevant to APP roles. In this respect, we define “tertiary care settings” (*German: Maximalversorgung*) as hospitals in which highly specialized medical services are provided, which are typically covered by university hospitals, academic teaching hospitals, or other forms of specialized hospitals. As we aim to conceptualize APP roles for the German tertiary care setting, all panel experts are required to speak German at an advanced level. All participants should be willing to participate in 3 survey rounds. Specific eligibility criteria are listed in Textbox 1.

Textbox 1. **Participant eligibility criteria.**

**Inclusion criteria**

- Physiotherapists presenting ≥3 years of work experience in tertiary care settings
- Physiotherapists holding leadership roles within physiotherapy departments in German tertiary care settings, including supervisors, team managers, and heads of physiotherapy departments
- Researchers with academic activities in tertiary physiotherapy care (educational and clinical)

**Exclusion criteria**

- Less than 3 years of clinical experience in tertiary care settings
- Physiotherapists holding leadership roles outside of the tertiary care setting
- Researchers with predominant academic activities in outpatient physiotherapy care

For successful recruitment of suitable experts, we will use the German network of researching physiotherapists of German university hospitals as this academic circle provides access to a diverse group of academic clinicians with established expertise and professional standing in the field. In the final report, we will describe recruitment and data collection procedures in accordance with the Checklist for Reporting Results of Internet E-Surveys [18]. In total, a sample size of 20 experts is targeted. This sample size was informed by availability of national experts and methodological recommendations suggesting that Delphi panels typically include 10 to 100 experts, depending on research scope and panel heterogeneity, as there is no agreed standard [19-21]. Following the experiences of Alt et al [22], who previously conducted a Delphi study with physiotherapy experts and reported a 39% response rate to study invitation, we plan to invite a minimum of 50 potentially eligible experts.

### Instrument Description and Data Collection Procedures

#### Overview

Data will be collected using online questionnaires (LimeSurvey) distributed via personalized invitation emails. Prior to commencing round 1, we will inform participants on data protection standards and efforts to ensure anonymity of study participation. As participants were purposively selected, no eligibility criteria will be queried prior to survey start. To enable matching of individual responses across the 3 Delphi rounds, participants will receive individualized survey links generated by the LimeSurvey token system. Participants will access each Delphi round through their personalized link. Participant responses will be pseudonymized at all times. The token list will only be accessible for the research team. After completion of the study, the token list will be deleted to prevent any potential reidentification. At this point, data will be completely anonymized.

#### Instrument Description of Round 1

In round 1, sociodemographic and occupational characteristics will be queried, including information about age, sex, clinical work experience, job positions, academic qualifications, advanced clinical training courses, and academic activities. Subsequently, participants will receive a theoretical input in which we will provide a definition of APP and a brief summary of the academic literature on the effectiveness, safety, and acceptance of APP roles outside of tertiary care settings. Similar to previous studies, this input serves as a “stimulus package” to ensure participants have a shared understanding of APP definitions and their internationally reported effectiveness [23].

The structure of the survey in round 1 will follow a predefined list of medical fields potentially relevant to APP roles in tertiary care. Specifically, this includes (1) emergency care; (2) internal medicine, pulmonology, or cardiology; (3) geriatrics or geriatric traumatology; (4) neurology or neurosurgery; (5) orthopedics or trauma surgery; (6) critical care; (7) oncology, hematology, or palliative care; (8) general surgery (visceral or thoracic surgery); (9) gynecology, obstetrics, or urology; (10) psychiatry or psychosomatic medicine; and (11) pediatrics.

For each medical field, participants will rate the relevance of implementing an APP role using a 7-point Likert scale ranging from “1=not relevant at all” to “7=very relevant.” If participants rate a medical field to be relevant (“5=fairly relevant,” “6=very relevant,” “7=extremely relevant”), we will ask them a set of open-ended questions to describe and explain practice areas, skills, and core competencies in the respective medical field. In that regard, the open-ended questions will be structured following the 4 key dimensions of the National Health Service (NHS) multiprofessional framework for advanced clinical practice (clinical practice, leadership and management, education, and research). If participants rate a medical field to be irrelevant (“1=not relevant at all,” “2=hardly relevant,” “3=slightly relevant,” “4=somewhat relevant”), we will ask them 1 open-ended question on reasons for exclusion. The final set of survey items of round 1 will depend on the number of practice areas, skills, and core competencies mentioned by participants. However, following the structure of round 1, a maximum number of 41 survey items will be administered.

#### Instrument Description of Round 2 and Round 3

In round 2, we will provide a structured synthesis of qualitative answers of open-ended questions of round 1 to participants. They will be able to review results of round 1 via a hyperlinked PDF file at the landing page of the survey. On the basis of this analysis, we will develop quantifiable survey items for each identified APP role, which describe and determine practice areas, skills, and core competencies. Within each APP role, developed items will be structured according to key domains of the NHS multiprofessional framework for advanced clinical practice. The relevance of each individual item will be queried using a 7-point Likert scale ranging from “1=not relevant at all” to “7=extremely relevant.” The number of survey items of round 2 will depend on results of Delphi round 1.

In round 3, we will provide results of survey round 2 in the same manner of the previous round via providing a hyperlinked PDF file at the landing page of the survey. Participants will then have the opportunity to confirm or adjust their responses from round 2 by reintroducing items with ambiguous response patterns. The results will be communicated to study participants via email. The number of survey items of round 3 will depend on the results of Delphi round 2.

**Figure 1.**
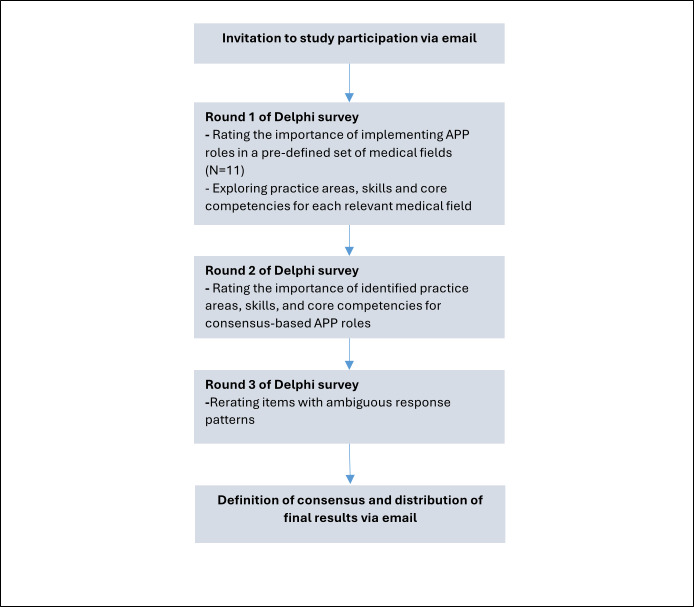
Methodological flow of the Delphi process. APP: advanced physiotherapy practice.

In total, the data collection period is planned to take 5 months. For each Delphi round, the data collection period will comprise 2 to 3 weeks. In each survey round, a reminder for survey participation will be sent at the end of week 2. Time between rounds will encompass 3 to 4 weeks. A summary of the methodological structure of this Delphi survey is provided in Figure 1.

### Data Analysis

In round 1, we will descriptively analyze close-ended survey items reporting absolute and relative frequencies of response options. Additionally, we will report measures of central tendency (median) and dispersion (IQR). In contrast, we will analyze open-ended survey items using a deductive-inductive thematic approach. In rounds 2 and 3, all close-ended survey items, including items that did not reach consensus, will be analyzed descriptively, reporting absolute and relative response frequencies as well as measures of central tendency and dispersion. Differences in item response patterns between rounds 2 and 3 will be analyzed using McNemar test statistics and Wilcoxon signed-rank test statistics. According to the study by Nasa et al [20], achieving response stability is a critical criterion for closing a Delphi study, often more important than consensus alone [20].

Across all rounds, we will export data to .csv files and analyze it using SPSS statistics (IBM Corp). We will additionally organize qualitative data of round 1 in .docx table formats and import them into a qualitative data management software (MAXQDA).

### Participant Attrition

Attrition across all Delphi rounds will be monitored and reported descriptively. The number of participants invited, responding, and completing each round will be documented. Dropouts will be reported for each round and, where appropriate, reasons for attrition will be explored descriptively. Only participants who completed a given round will be invited to the subsequent round. Dropouts will not be replaced.

### Measurement of Consensus

As the Delphi method involves several rounds of discussion and different expert opinions, reaching 100% consensus is in most cases an ambitious and impractical goal. For this study, an a priori consensus threshold of ≥70% agreement per item (ratings of “5=fairly relevant,” “6=very relevant,” “7=extremely relevant”) was defined. Items achieving at least 70% agreement will be retained for the final set. Items receiving 30% agreement or less will be excluded from further consideration. Items falling between 31% and 69% agreement will be reintroduced in the subsequent round (round 3) to allow participants to reconsider their ratings in light of the group’s feedback. This procedure of consensus measurement has previously been applied and is in line with the studies by Kundakci et al [24] and Heuzenroeder et al [19]. Items still falling between 31% and 69% agreement after round 3 is completed will be excluded from the final list of items.

### Ethical Considerations

Ethics approval (number 2025‐11) was obtained from the ethics committee of the German Federal Association for Physiotherapy (*Physio Deutschland e.V., Köln GER*).

Efforts will be made to minimize participant burden throughout the Delphi process. Participants will be informed about the expected number of rounds prior to enrollment. Invitations to participate will be sent via email, including information sheets on study objectives, procedures, informed consent requirements, and data protection standards. Data will be collected in a pseudonymized form, hindering direct conclusions about participant identities. Participation will be voluntary. Reminder emails will be used sparingly to maximize response rates while avoiding excessive contact. In addition, survey rounds will be designed to be as concise as possible with subsequent rounds including only items that tend to reach consensus.

## Results

Data collection took place between January and May 2026. In round 1 of this study, a total of 98 participants were invited. We received a total of 56 responses, of which 37 fully completed the survey. For round 2, only participants who fully responded to round 1 were invited (n=37). Among them, 34 completed the second Delphi round and were invited for round 3. Data collection for the third and final round was completed in May 2026 and included 32 final respondents. The manuscript reporting on results of this Delphi survey is planned to be submitted to a peer-reviewed scientific journal in August 2026.

## Discussion

### Expected Findings

We anticipate that this Delphi study will yield expert consensus on practice areas in which APP roles are most urgently needed within German tertiary care settings. In addition, the study is expected to generate a structured set of competencies across the domains of clinical practice, leadership and management, education, and research, aligned with international frameworks for advanced practice. By synthesizing the perspectives of academic and clinically experienced physiotherapists, the findings will provide a context-specific competency profile that reflects both the demands of tertiary care and the evolving role of physiotherapists in multidisciplinary health care teams. Specifically, by defining clear competencies and roles, the study addresses common implementation barriers of APP roles into clinical settings [25].

While international frameworks such as the NHS multiprofessional advanced practice framework provide a useful structure, their transferability to the German health care context is limited due to differences in regulatory and professional structures (eg, scope of practice and role recognition). Therefore, it is used as a conceptual reference, and its referral to German legislative conditions needs to be interpreted cautiously.

Beyond identifying priority areas and competencies, the results are expected to have several practical implications. They may inform the development of education and training curricula for advanced physiotherapy roles and may support policy discussions on role definition and professional regulation in Germany. In this way, the study aims to contribute to a structured and context-sensitive development of APP roles within the German health care system. Moreover, the results are expected to highlight areas where APP roles may strengthen patient care pathways, address projected workforce shortages, and contribute to the professional development and attractiveness of physiotherapy in Germany. While consensus is unlikely to cover all potential practice areas exhaustively, we expect the results to provide a robust foundation for further empirical research, pilot implementation projects, and health policy discussions on the integration of APP roles into the German health care system.

### Study Limitations

This study is subject to several potential limitations. First, the purposive sampling strategy may introduce selection bias and limit the representativeness of the expert panel, particularly if certain professional roles or specialties are overrepresented. Second, participant attrition across multiple rounds poses a risk to the robustness of findings, as dropouts may bias consensus toward the perspectives of those who complete all survey rounds. Third, although the a priori consensus threshold is informed by previous Delphi studies in health sciences, it remains to some extent arbitrary. Fourth, the method relies on subjective judgments of experts, which, although informed by clinical and academic experience, may not fully capture the complexity of practice realities. Finally, as the study is conducted within the German tertiary care context, generalizability to other health care systems or care levels may be limited.

### Dissemination and Future Directions

The findings of this study will be disseminated through peer-reviewed publications, conference presentations, and active engagement with relevant professional and policy stakeholders. Future research may focus on validating and shaping identified tasks and core competencies, exploring implementation strategies, and evaluating the impact of APP roles in tertiary clinical practice. Additionally, the findings will be distributed to all participants via email.

### Conclusions

This Delphi study is expected to provide a structured and consensus-based foundation for defining APP roles in German tertiary care. By identifying priority practice areas and core competencies, the study will contribute to a more distinct conceptualization of APP. The findings may serve as a basis for the development of targeted education and training programs, inform profession and policy discussions, and support the design of future implementation initiatives. Ultimately, this work aims to support the systematic and context-specific integration of APP roles into German tertiary care.

## Abbreviations

APN: advanced practice nurse
APP: advanced physiotherapy practice
NHS: National Health Service
